# Impact of ESG performance of China’s listed energy companies on their going global performance

**DOI:** 10.1038/s41598-026-54897-z

**Published:** 2026-05-27

**Authors:** Xuanhao Huang, Dan Liu, Yueming Sun

**Affiliations:** 1 School of Energy and Mining Engineering, China University of Mining and Technology-Beijing, Beijing, 100083 China; 2 School of Management, China University of Mining and Technology-Beijing, Beijing, 100083 China; 3 Finance Bureau of Huangpu District, Guangzhou, 510530 China; 4 College of Geoscience and Surveying Engineering, China University of Mining and Technology-Beijing, Beijing, 100083 China

**Keywords:** ESG performance, Internationalization performance, Energy sector, Corporate reputation, Green innovation, Business and management, Business and management, Economics, Economics, Environmental social sciences, Finance, Finance

## Abstract

The energy sector stands as the foremost battleground for Chinese companies seeking to expand overseas. It is worth investigating whether listed energy companies can leverage their ESG performance to forge new competitive advantages in the international market, navigate the high standards and stringent regulations of the global arena, and propel them to go global. Using panel data of 1,882 firm-year observations of Chinese A-share listed energy companies from 2013 to 2023, this study examines the association between ESG performance and internationalization performance measured by overseas revenue. Fixed-effects regression models are employed to control for unobserved heterogeneity. The main findings are as follows: (1) ESG performance is positively and significantly associated with internationalization performance (*β* = 0.7816, *p* < 0.01), and the result remains robust across multiple robustness checks. (2) Mechanism analysis shows that ESG performance enhances internationalization outcomes by improving corporate reputation (*β* = 0.5045, *p* < 0.01) and promoting green innovation capability (*β* = 0.3740, *p* < 0.01). (3) Heterogeneity analysis indicates that the positive association is stronger for state-owned energy companies and companies operating in the new energy segment and in the non-Belt and Road Initiative countries. These results provide quantitative evidence that ESG practices are closely linked to international expansion outcomes in the energy sector. The policy implications are that regulators may strengthen standardized ESG disclosure and verification to reduce information asymmetry in cross-border markets. Companies can embed ESG governance into overseas compliance and green innovation strategies to enhance internationalization outcomes.

## Introduction

**Fig. 1:**
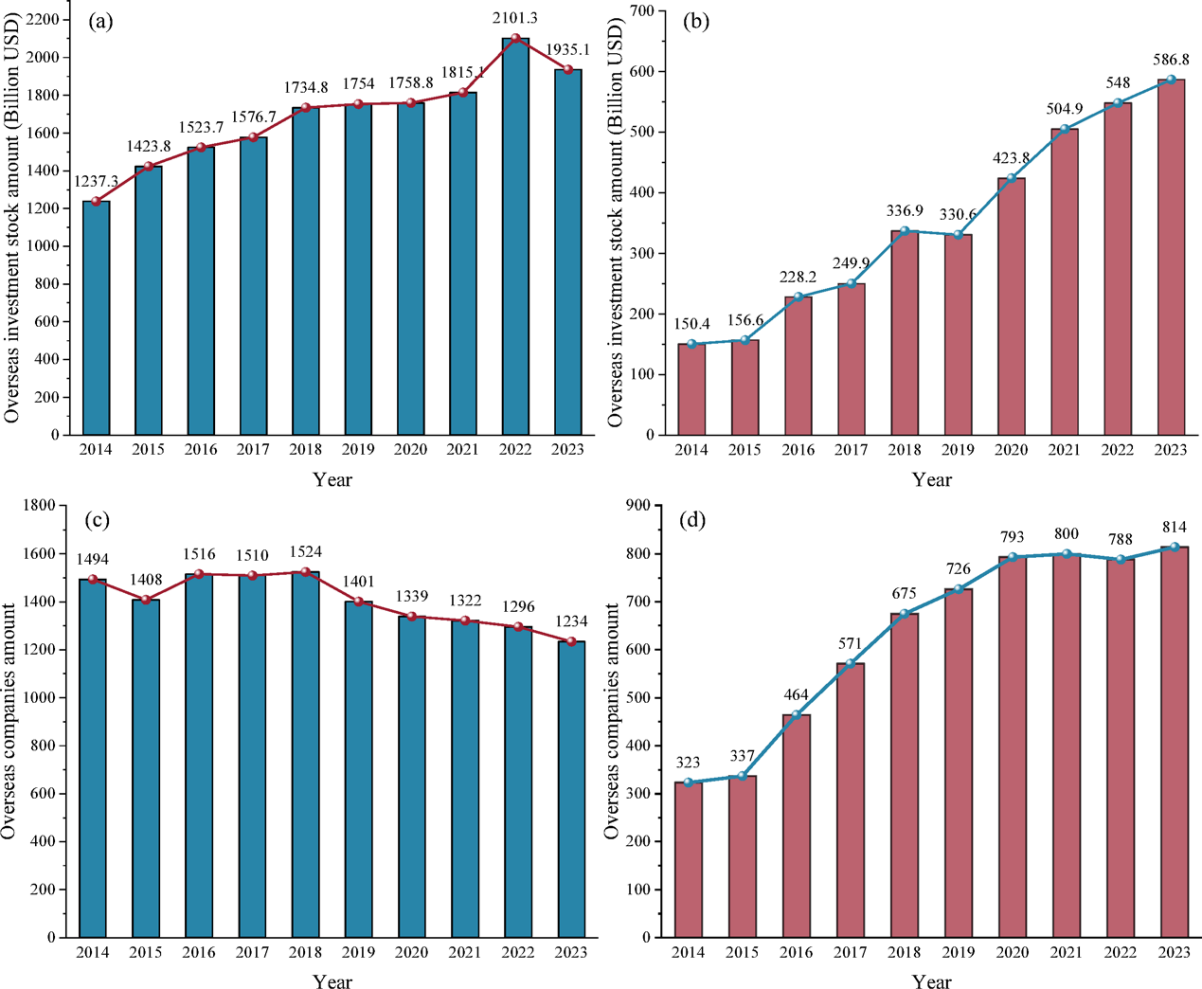
Overseas investment trends of China energy industry: (**a**) and (**b**) are respectively the overseas investment stock amounts of mining companies and electric power related companies, (**c**) and (**d**) are respectively the overseas companies amounts of mining industry and electric power related industry.

Global environmental governance faces severe challenges. Issues like environmental pollution, resource depletion, and ecological imbalance are growing increasingly acute^1–3^. Advancing sustainable economic and social development by addressing these issues has become a critical global priority. The Environmental, Social, and Governance (ESG) framework, as a key tool for coordinating economic, environmental, and social development, has drawn widespread attention from academia and practitioners^4–6^. Global statistics show over 1,000 ESG-related policies and regulations, reflecting a growing trend toward stronger ESG information disclosure^7–9^. ESG performance has become essential for companies pursuing globalization expansion (“going global”). Chinese companies face anti-globalization trends, international trade protectionism, new ESG regulations, and green trade barriers^10,11^. This complex landscape presents significant challenges totheir “going global”. The energy sector plays a critical role, with its export economic value maintaining sustained high growth^12,13^. Figure 1 illustrates China’s energy industry overseas investment trends from 2014 to 2023. *China’s Energy Transformation Vision 2024* outlined the latest developments in energy transformation: strengthening international cooperation, building energy partnerships, co-developing the green Belt and Road Initiative, constructing large-scale energy infrastructure, supplying new technologies and equipment for global energy transformation, and fulfilling China’s role as a major contributor to global energy transformation. However, the energy industry faces inherent ESG governance risks^14^. China provides a salient setting as its listed energy companies are increasingly active in cross-border operations while simultaneously facing rapidly evolving ESG disclosure practices and external green trade barriers. Exploring and studying the impact of ESG performance on the “going global” performance of listed energy companies is of significant practical importance.

Current academic perspectives on ESG’s impact on companies’ “going global” focus on two main views: “positive impact” and “U-shaped impact” relationships^15–17^. The former perspective primarily draws on stakeholder theory and resource-based theory. It maintains that ESG helps companies meet stakeholder expectations. It also enhances company reputation and helps companies obtain more resources. These actions form competitive advantages to promote “going global”^18,19^. Moreover, ESG advantages add significant value in outward investment. They enable access to low-cost debt capital and favorable financing^20^. All these benefits facilitate market integration, regulatory compliance, and sustainable international operations. The other view argues that company ESG performance doesn’t have a simple positive linear effect on “going global” performance; instead, it shows a non-linear relationship^21–23^. Improving environmental (E) performance to meet international market demands effectively boosts competitiveness, promoting “going global” performance. Conversely, improving social (S) and governance (G) performance requires initial high costs for companies. This often stifles short-term “going global” growth. Yet good ESG performance still benefits “going global” performance over time.

Companies pursuing global expansion face significant external challenges, including information asymmetry, capability deficits, and insufficient competitiveness^10,24^, which collectively heighten survival risks in international markets. Global sustainable development requirements are intensifying, driving progressive unification of ESG information disclosure standards. In this context, good ESG performance has become a key competitive advantage driving companies’ “going global”. Current academic research on the impact of companies’ ESG performance on “going global” remains nascent. Many dimensions remain unexplored. No definitive consensus exists on how ESG influences “going global” through specific mechanisms. Most existing research draws samples from all listed companies, potentially obscuring industry-specific nuances. However, significant variations may exist in the ESG and “going global” performance relationship across industries. Few studies conduct targeted industry-level analyses.

The above analysis highlights two key contexts: China’s deep implementation of the “going global” strategy, and the global emphasis on sustainable development. This study conducts an in-depth exploration of ESG performance’s role and impact pathways in listed energy companies’ “going global” processes. This study addresses the following research questions: (1) Does ESG performance improve the “going global” performance of listed energy companies in China? (2) Through which mechanisms — corporate reputation and green innovation — does ESG affect “going global” performance? (3) Does the ESG — “going global” link vary by ownership types, energy types and host markets? It centers on the needs of global energy and green development, with empirical analysis rooted in the energy industry’s “going global” practices. These findings will offer actionable development strategies for listed energy companies to enhance their “going global” performance through ESG practice. The contributions of this paper are: (1) The impact of ESG performance on the “going global” performance of China’s listed energy companies is studied for the first time, filling industry-specific evidence; (2) Companies ESG performance can significantly boost China’s listed energy companies’ “going global” performance; (3) Company reputation and green innovation have mediation effect, indirectly improving the “going global” performance; (4) State-owned, new energy and non-Belt and Road Initiative companies have stronger sensitivity on ESG performance.

The structure of this paper is as follows: Section “Theoretical analysis and research hypotheses” is based on theoretical analysis, which proposes research hypotheses. Section “Data, variables, econometric models” provides a detailed introduction to the data and variables used and constructs econometric models. In Section “Empirical results and analysis”, we mainly describe and analyze the empirical results, including robustness tests, mechanism analysis and heterogeneity analysis. Lastly, “Discussion and implications”, “Limitations and future research” and “Conclusions” are presented.

## Theoretical analysis and research hypotheses

### Company ESG performance and “going global” performance

Previous research has confirmed that ESG performance plays a critical role for companies pursuing global expansion^9,25^. Drawing on company reputation theory, companies build their reputation through good ESG performance to support international market expansion^26^. This reputation then enhances product reputation, boosting international market acceptance and significantly improving “going global” performance. The sustainable development concept is now globally entrenched. The core tenets and connotation of ESG require companies to regulate operations across three dimensions: environmental protection, social responsibility, and company governance. These practices help “going global” companies reduce compliance costs, mitigate regulatory risks, and strengthen social reputation, providing incremental gains for “going global” performance^19^. Good ESG advantages reduce regulatory sanction threats and litigation risks, easing international market access^27^. Timely ESG reporting by companies transmits key signals and strengthens stakeholder communication^28^. Good ESG performance builds trust with stakeholders, thereby improving “going global” performance^29^. Nationally, governments have recently tightened requirements for companies to follow sustainable development principles and enhance capabilities. Internationally, importers prioritize partners’ ESG performance as a key selection factor^30^. They prefer collaborating with companies with excellent ESG performance, which in turn improve importers’ own ESG performance^31^. Thus, good ESG performance emerges as a critical determinant of companies’ “going global” performance. Therefore, based on this theoretical analysis, we propose Hypothesis 1.

#### Hypothesis 1

ESG performance can help promote and improve the “going global” performance of companies.

### Mechanism effect of company reputation and green innovation

#### ESG and company reputation

Prior studies suggest that ESG acts as a credible signal that strengthens company reputation and stakeholder trust, which can facilitate market access and international revenue generation^32–34^. Reputation theory suggests that companies’ good ESG performance enhances their reputation, emphasizing brand value and image^35^. This expands international markets through competitive advantages, ultimately boosting their “going global” performance. Global consumer decisions are influenced by sustainable development principles^36^. These trends require companies to strengthen ESG performance. ESG mandates environmental commitment: energy conservation, emission reduction, and ecological protection. In social responsibility, companies need to actively engage in public welfare and prioritize stakeholder needs^37,38^. In terms of corporate governance, companies build robust compliance mechanisms^39^. These three dimensions work together to achieve coordinated, sustainable long-term development, demonstrating the correctness of company value orientation and strong business reputation^40^. For Chinese listed energy companies, ESG concepts play a key guiding role in their “going global” efforts. These concepts prompt companies to focus more on improving clean production methods, building green supply chains, managing carbon footprints, and providing low-carbon energy solutions^41^. This supports local economic growth in host countries. For companies themselves, it helps meet sustainability goals and strengthens company image building. These effects generate spillover or halo effects, earning recognition from local governments, investors, and other stakeholders^31^, which ultimately enhances companies’ reputation.

ESG performance conveys critical operational signals^42,43^. It reduces information asymmetry and deepens stakeholder understanding of companies’ sustainability efforts. These proactive signals deliver commercially valuable, reliable business information to stakeholders, which helps companies build a strong company reputation^3^. Additionally, company reputation reflects societal evaluations. Strong company reputation boosts stakeholder confidence in companies’ sustainable development. For host-country companies, company reputation is a key factor when selecting foreign partners for long-term collaboration^28^. They are more likely to select high-reputation companies. This tendency enhances customer satisfaction and loyalty, thereby strengthening the companies’ competitive edge in global markets. As companies “go global”, they develop a reputation-based competitive edge, securing sustained advantages. This ultimately enhances their “going global” performance. Based on the above analysis, we propose Hypothesis 2.

##### Hypothesis 2

Good ESG performance can enhance company reputation in companies, thereby improving their “going global” performance.

#### ESG and green innovation

Sustainable development theory defines green innovation as the innovative process through which companies develop or improve technologies, processes, products, services, and management to reduce environmental harm and enhance resource efficiency^44,45^. ESG ratings and green innovation both reflect the “win-win” principle of sustainable development^46^. ESG performance contributes to companies’ “going global” performance by driving green innovation^47^. The three dimensions of ESG directly enable green innovation. Environmental (E) innovations generate energy-saving and pollution-reduction benefits, while social (S) innovations fulfill stakeholder responsibilities. In global markets, these innovations align with host countries’ environmental protection policies, laws, regulations, and strict “hard constraint” standards. This compels companies to adopt measures to transform and upgrade production processes, technologies, and marketing methods, thereby enabling companies to meet environmental requirements and achieving green innovation to adapt to global markets^48^. Consequently, ESG performance is shown to drive companies toward green innovation. By enabling green-development competitive advantages globally and enhancing sustainable development capabilities^49^, ESG ultimately improves companies’ “going global” performance.

Company ESG advantages enhance green innovation capabilities. Actively fulfilling social responsibility strengthens companies’ trust and stakeholder support, providing critical resources for technological innovation^50^. This trust and support translate into institutional backing, including tax incentives, fiscal subsidies, and policy support, which lowers innovation costs and elevates innovation capabilities. Meanwhile, robust company governance guides management to prioritize environmental performance, driving green innovation^51^. Enhancing production processes and independent innovation capabilities further improves export quality, better meeting international market demands and expanding market share^52^. Consequently, this transformation from “going out” to “going in” manifests as good “going global” performance. Based on this analysis, we propose Hypothesis 3.

##### Hypothesis 3

Good ESG performance can promote green innovation in companies, thereby improving their “going global” performance.

**Fig. 2:**
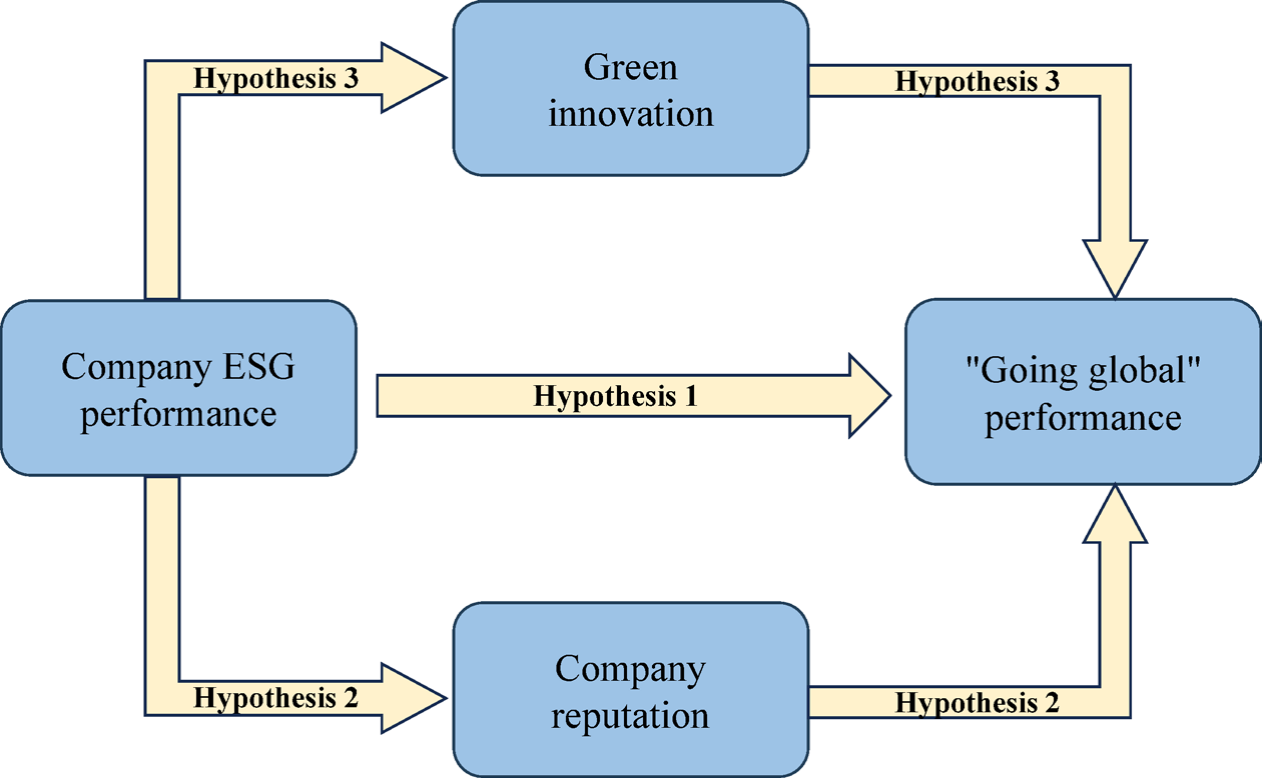
Theoretical model.

We propose the theoretical model, based on the above hypotheses, as shown in Fig. 2.

## Data, variables, econometric models

### Sample selection and data sources

Current academic research on the energy industry has yielded abundant results. However, the definition and scope of listed energy companies remain inconsistent across studies. To address this, this study adopts the classification and definition methods for the energy industry from Tang et al.^53^ to select research samples. It also aligns with the *Shanghai Stock Energy Index Compilation Plan* and uses the industry classification criteria of the China Securities Index (CSI) to identify listed energy companies as the research objects, as detailed in Table 1.

**Table 1:** Sample section of energy sector.

Energy category	Details	Industry segmentation of CSI Index
Electric power	Thermal power	Thermal power generation
	Power grid	Power grid self-control equipment; Manufacturing of wires and cables; Power transmission and transformation
Nuclear energy	Nuclear power	Nuclear power materials and equipment; Nuclear power generation
Coal	Coal	Coal mining equipment; Coal mining and washing
Oil and gas	Oil	Oil production and sales
	Gas	Gas production and sales
New and renewable energy	Hydropower	Hydropower generation
	Wind energy	Wind power equipment; Wind power generation
	Light energy	Photovoltaic raw materials; Photovoltaic cell module; Photovoltaic system engineering
	Other energy	Producers using other energy sources, including biogas, biomass energy, geothermal energy, tidal energy

The data used in this study are primarily sourced from the CSI, which categorizes the energy industry into four distinct groups and provides sample company codes for listed energy companies. Overseas business income and other relevant variable data are extracted from the CSMAR and Wind databases. Additionally, ESG scores for listed energy companies are obtained from the Huazheng ESG Database. Data processing and matching were conducted using Excel and Stata 18.5. To ensure research validity, data screening is performed based on the following criteria: (a) to exclude samples with missing variable data; (b) to exclude ST and *ST companies; and (c) to perform 1% winsorization on both ends for all continuous variables.

### Variables definition

In this paper, the explained variable is the “going global” performance of listed energy companies. This performance metric evaluates the profit value derived from their business activities in other countries and regions over a specific period. Most existing studies quantify this performance using overseas income indicators^9,39,54^. Drawing on the methodology of Liu et al.^54^, this study uses the companies’ overseas income as a proxy variable for their “going global” performance. The explanatory variable is company ESG performance. As a comprehensive indicator measuring a company’s ESG practices, ESG performance has a well-defined evolution trend. Numerous institutions and organizations have invested in ESG rating data collection, leading to a large number of existing ESG rating agencies. These primarily include Huazheng, CNRDS, and Bloomberg ESG databases. In this paper, we consider the relationship between the ESG governance capability of the parent company and its overseas business performance, rather than comparing it with the ESG advantages of peer companies in overseas countries. Following the approach of Xie and Lyu^55^, this paper selects the Huazheng ESG database. We assign scores ranging from 1 to 9 to the nine grades (C to AAA) of company ESG ratings: Grade C is assigned a score of 1, Grade CC a score of 2, and so on, with subsequent grades (CCC to AAA) assigned scores of 3 to 9. The higher the score, the better the company ESG performance. We control for SIZE, LEV, TAT, TOP10, GRO, FAS, OVE, and AGE because these factors are commonly associated with companies’ international revenue capacity through scale economies, financing constraints, operational efficiency, governance, and managerial international experience. The definitions and detailed descriptions of all variables are presented in Table 2.

**Table 2:** Variables definition.

Type	Name	Symbol	Description
Explained	“Going global” performance	Overseas	Logarithmic calculation of annual overseas revenue for companies
Explanatory	Company ESG performance	ESG	The data comes from Huazheng ESG Score Database
Control	Total asset turnover	TAT	Operating revenue / [(total initial assets + total ending assets) / 2]
	Ownership concentration	TOP10	Number of shares held by the top 10 shareholders / total shares
	Company size	SIZE	Logarithmic calculation of total assets of the company at the end of the year
	Fixed assets proportion	FAS	Fixed asset to total asset ratio
	Revenue growth rate	GRO	(Total operating revenue for this year - total operating revenue for previous year) / operating revenue for previous year
	Number of directors with overseas work experience	OVE	Add 1 to the number of directors working overseas experience and take the natural logarithm
	Asset liability ratio	LEV	Total year-end liabilities divided by total year-end assets
	Company age	AGE	Take the natural logarithm of the establishment period of the company
Intermediary	Green innovation	GRE	The research method of Wang and Wang^56^ is referred. Independent patent applications for green innovation in companies are counted to measure their green innovation capabilities. Add 1 to the number of green patent applications and take the natural logarithm.
	Company reputation	REP	The research method of Li and Feng^57^ is referred. The number of positive reports obtained by companies in newspapers and online reports each year add 1, and then taking the logarithm. It is used for the measure of company reputation.

### Model construction

To explore and examine the impact of ESG performance of listed energy companies on their “going global” performance, this study constructs a fixed effects regression model with both year and industry-specific fixed. The model specification is detailed as follows:

1 $$Oversea{s_{i,t}}={\alpha _0}+{\alpha _1}ES{G_{i,t}}+Contro{l_{i,t}}+In{d_{i,t}}+Year+{\varepsilon _{i,t}}$$

where *i* represents the company; *t* represents the year; *Overseas*_*i, t*_ represents *i* company’s “going global” performance in *t* year; *α*_0_ is constant term; *Control*_*i, t*_ represents the control variable; *ESG*_*i, t*_ represents *i* company’s ESG performance in *t* year; *Year* represents the year fixed effects; *Ind* represents the industry-specific fixed effects; *ε*_*i, t*_ represents stochastic disturbance term. We focused on regression coefficients *α*_1_. If the *α*_1_ is significantly positive, the empirical results will support our theoretical analysis.

The mediation effect model is constructed, referring to the research of Wen and Ye^58^. We use the three-step method, as follows:

- Company reputation mechanism (REP).

2 $$RE{P_{i,t}}={\alpha _0}+{\beta _1}ES{G_{i,t}}+Contro{l_{i,t}}+In{d_{i,t}}+Year+{\varepsilon _{i,t}}$$

3 $$Oversea{s_{i,t}}={\alpha _0}+{\beta _1}ES{G_{i,t}}+\beta _{1}^{\prime }RE{P_{i,t}}+Contro{l_{i,t}}+In{d_{i,t}}+Year+{\varepsilon _{i,t}}$$

b. Green innovation mechanism (GRE).

4 $$GR{E_{i,t}}={\alpha _0}+{\gamma _1}ES{G_{i,t}}+Contro{l_{i,t}}+In{d_{i,t}}+Year+{\varepsilon _{i,t}}$$

5 $$Oversea{s_{i,t}}={\alpha _0}+{\gamma _1}ES{G_{i,t}}+\gamma _{1}^{\prime }GR{E_{i,t}}+Contro{l_{i,t}}+In{d_{i,t}}+Year+{\varepsilon _{i,t}}$$

## Empirical results and analysis

### Baseline regression

Using the fixed effects regression model, this study performed a baseline regression analysis with the explained variable (Overseas) and explanatory variable (ESG). The empirical results are presented in Table 3. The findings reveal that ESG positively and significantly affects the “going global” performance of listed energy companies, thereby supporting Hypothesis 1. Specifically, the coefficient of ESG is 0.7816, which is significant at the 1% level. This empirical analysis demonstrates that good ESG performance contributes to enhancing companies’ “going global” performance.

**Table 3:** Analysis results of baseline regression.

Variables	Overseas
ESG	0.7816***
	(5.6843)
TAT	0.9187***
	(5.2437)
TOP10	0.1791
	(1.1097)
SIZE	4.0058***
	(4.3727)
FAS	3.8871***
	(8.6592)
GRO	0.3635*
	(1.7501)
OVE	-0.0185*
	(-1.9474)
LEV	1.8426***
	(6.6520)
AGE	0.5358***
	(8.2178)
Constant	10.3625***
	(9.1720)
Year	Control
Ind	Control
R-squared	0.3326
N	1882

*, **, and *** indicate 10%, 5%, and 1% significance levels, respectively, the values of t are in parentheses, the following table is the same.

### Robustness tests and endogeneity issues

#### Change of sample period

The COVID-19 pandemic exerted an unprecedented impact on global economic operations. During the crisis, the international trading system experienced severe disruptions, characterized by supply chain fragmentation, logistics paralysis, and demand contraction. Companies faced numerous challenges, including order cancellations, soaring transportation costs, and delivery delays. These external shocks, exceeding the magnitude of normal economic fluctuations, distorted the normal “going global” performance of companies. Typically, the impact of ESG performance on “going global” is exerted through long-term, gradual mechanisms in relatively stable market environments. However, the pandemic-induced shocks obscured the mild ESG impact path. Therefore, including such disrupted data would interfere with accurately judging the intrinsic relationship between ESG performance and “going global”. To address this, following the method of Wang and Bu^59^, this study adjusted the sample period by shortening the time window. We retained only data from 2013 to 2019 for the robustness test.

The results of robustness test for changing the sample period are shown in Table 4 Column (1). ESG of companies and their “going global” performance still show a positive correlation at the 1% level, after excluding the pandemic data. The results indicate that good ESG performance has a positive impact on the “going global” performance of listed energy companies. This is consistent with the baseline regression results, further supporting Hypothesis 1.

#### Substitution of explanatory variable

The method of replacing explanatory variable is adopted for robustness test, referring to the research of Xie and Lyu^55^. We replaced the Huazheng rating data with Bloomberg ESG rating data (ESG_2). Huazheng and Bloomberg as different rating agencies have their own data collection, analysis, and rating systems. Single data sources may have biases or systematic errors. The data source was changed, which can avoid these errors and make the empirical results more convincing. Bloomberg ESG rating was used to replace Huazheng rating for robustness test, simulating an external market perspective on company ESG performance. The credibility of research results is enhanced.

Table 4 Column (2) shows the test results after replacing the explanatory variable. It indicates that regardless of which ESG rating data are used, there is a positive correlation between the explanatory variable and the explained variable, which is significant at the 1% level. This indicates that the research results are robust.

**Table 4:** Robustness analysis results: change of sample cycle and substitution of explanatory variables.

Variables	(1)	(2)
	Overseas	Overseas
ESG	0.3513***	
	(2.9422)	
TAT	0.8591***	0.8102***
	(2.9768)	(4.4590)
TOP10	0.3644	0.4879***
	(1.5843)	(2.8990)
SIZE	4.0464***	2.5069***
	(3.3575)	(4.2411)
FAS	4.7977***	3.4571***
	(7.6690)	(5.8864)
GRO	0.2820	0.3312
	(1.0100)	(1.4766)
OVE	-0.0081	-0.0103
	(-0.5870)	(-1.0198)
LEV	1.9355***	1.1080***
	(4.5169)	(3.2684)
AGE	0.5672***	0.2432***
	(8.0454)	(3.7130)
ESG_2		0.0485***
		(9.7000)
Constant	12.5792***	16.2756***
	(11.2656)	(29.5490)
Year	Control	Control
Ind	Control	Control
R-squared	0.3217	0.4036
N	832	1652

#### Propensity score matching method

The model may also face sample self-selection bias due to randomness. This paper uses propensity score matching (PSM) to address this issue. We re-ran the regression model with filtered samples. First, we calculated the median ESG performance score. The original sample was split into treatment and control groups. Control variables were included as covariates. Next, 1:1 nearest neighbor matching was applied. Finally, we re-conducted the regression analysis post-matching. Results are shown in Table 5, with Column (1) as the baseline regression. Column (2) presents regression results using PSM to reduce sample size and exclude unmatched samples. This reduced unmatched samples by 86%, minimizing self-selection bias. Results confirm a significant positive impact of ESG performance on listed energy companies’ “going global” performance at the 1% level, supporting the baseline conclusion.

**Table 5:** Robustness analysis results of propensity score matching method.

Variables	(1)	(2)
	Overseas	Overseas
ESG	0.7816***	0.6939***
	(5.6843)	(3.7087)
TAT	0.9187***	0.8973***
	(5.2437)	(3.9933)
TOP10	0.1791	0.1979
	(1.1097)	(0.7620)
FAS	4.0058***	4.3755***
	(4.3727)	(3.7574)
SIZE	3.8871***	3.5405***
	(8.6592)	(5.5174)
GRO	0.3635*	0.6233**
	(1.7501)	(2.4806)
OVE	-0.0185*	-0.0418***
	(-1.9474)	(-2.8435)
LEV	1.8426***	1.7995***
	(6.6520)	(3.0208)
AGE	0.5358***	0.5238***
	(8.2178)	(4.4693)
Constant	10.3625***	9.8693***
	(9.1720)	(6.3842)
Year	Control	Control
Ind	Control	Control
R-squared	0.3326	0.3210
N	1882	1796

#### Hysteresis effect

The baseline regression results show that the better a company’s ESG performance, the more prominent its “going global” performance. However, the interaction between the two needs consideration. Companies with good “going global” performance may more actively enhance their ESG performance, which could lead to endogeneity issues due to mutual causality. This paper referrs to the research of Liu et al.^60^. To reduce potential reverse causality in the model, we conducted a robustness test on the explanatory variable using ESG lagged by one period. We included ESG lagged by one period as the explanatory variable in the model, with results shown in Table 6. Results indicate that when ESG is lagged by one period, it remains significantly positive at the 1% level. This suggests that good ESG performance still positively promotes companies’ “going global” performance after accounting for reverse causality, supporting the reliability of the baseline results.

**Table 6:** Robustness analysis results of hysteresis effect.

Variables	Overseas
LESG	0.6840***
	(3.8930)
Control variables	YES
Year	Control
Ind	Control
R-squared	0.237
N	1693

### Mechanism analysis

#### Company reputation

Hypothesis 2 requires verification: whether good ESG performance enhances company reputation and thereby improves “going global” performance. This paper estimated a regression model for company reputation using the REP measurement index, incorporating control variables and double fixed effects (year and industry-specific). Table 7 presents the regression results. Column (2) shows the impact of ESG on company reputation: a 1-unit increase in ESG leads to a 0.5045 improvement in company reputation, significant at the 1% level. Results indicate that listed energy companies emphasize ESG performance, aligning with the international “carbon reduction” trend amid the current accelerated global energy transition. By disclosing ESG information prioritized by the international market, these companies enhance their ESG ratings, eliminate the traditional high-energy image, and build a positive reputation.

Constructing the mechanism model, this study aimed to verify how company reputation mediates the effect of ESG performance on listed energy companies’ “going global” performance. Results are presented in Table 7. Column (1) reports a coefficient of 0.7816 between ESG and Overseas, statistically significant at the 1% level under the double fixed effects model. Column (3) shows a coefficient of 0.6077, still statistically significant at the 1% level, after including the company reputation indicator. Comparing Columns (1) and (3), the coefficient decreases from 0.7816 to 0.6077. This decline occurs because part of ESG performance’s impact on “going global” performance is channeled through company reputation. This suggests that some of ESG performance direct impacts on “going global” performance, with the rest indirect via reputation enhancement. Column (3) further shows the coefficient of company reputation on “going global” performance is 0.3215, which is statistically significant at the 1% level. This confirms company reputation acts as a mediator between ESG performance and “going global” performance. Put differently, good ESG performance acts as a “reputation key”, helping listed energy companies unlock trust in international markets, thereby promoting their “going global” and improving performance.

**Table 7:** Mechanism analysis results of company reputation.

Variables	(1)	(2)	(3)
	Overseas	REP	Overseas
ESG	0.7816***	0.5045***	0.6077***
	(5.6843)	(6.4763)	(4.6999)
REP			0.3215***
			(9.9228)
TAT	0.9187***	0.3840***	0.9273***
	(5.2437)	(3.6364)	(5.4323)
TOP10	0.1791	-0.0870	0.2843
	(1.1097)	(-1.3083)	(1.5211)
SIZE	4.0058***	1.7415***	3.2537***
	(4.3727)	(4.1395)	(3.0169)
FAS	3.8871***	0.1163	3.9200***
	(8.6592)	(0.5850)	(7.8166)
GRO	0.3635*	-0.0011	0.3440
	(1.7501)	(-0.0147)	(1.6120)
OVE	-0.0185*	-0.0145**	-0.0089
	(-1.9474)	(-2.3387)	(-0.8812)
LEV	1.8426***	1.0188***	1.3846***
	(6.6520)	(4.7189)	(4.4096)
AGE	0.5358***	0.0592	0.5132***
	(8.2178)	(1.2569)	(8.2112)
Constant	10.3625***	-1.3212*	10.6721***
	(9.1720)	(-1.9098)	(9.6171)
Year	Control	Control	Control
Ind	Control	Control	Control
R-squared	0.3326	0.6020	0.3657
N	1882	1882	1882

#### Green innovation

The impact of ESG on “going global” performance is partially mediated by green innovation, which is indicated by the regression results, as shown in Table 8. Column (1) shows ESG directly and significantly affects “going global” performance, with a coefficient of 0.7816 and significant at the 1% level. This indicates good ESG performance directly drives companies’ “going global” performance improvement. Column (2) shows the coefficient ESG on green innovation is 0.3740, which is significant at the 1% level, suggesting companies with good ESG performance invest more in and achieve greater success in green innovation. In Column (3), after including green innovation, the coefficient of ESG on “going global” performance drops to 0.6692 but remains significant at 1% level. Meanwhile, green innovation has a significantly positive impact on “going global” performance, with a coefficient of 0.1820, confirming it acts as a mediator between ESG performance and “going global” performance. Good ESG performance directly enhances companies’ “going global” performance, while indirectly strengthening their competitiveness and attractiveness in international markets by fostering green innovation. Overall, green innovation is a key channel through which ESG promotes the growth of companies’ “going global” performance, highlighting the dual benefits of sustainable development strategies.

**Table 8:** Mechanism analysis results of green innovation.

Variables	(1)	(2)	(3)
	Overseas	GRE	Overseas
ESG	0.7816***	0.3740***	0.6692***
	(5.6843)	(8.5000)	(4.9792)
GRE			0.1820***
			(3.5616)
TAT	0.9187***	-0.0130	0.9677***
	(5.2437)	(-0.1605)	(5.6957)
TOP10	0.1791	-0.2340***	0.2011
	(1.1097)	(-7.3585)	(1.2100)
SIZE	4.0058***	1.4689***	3.8333***
	(4.3727)	(5.4687)	(3.9993)
FAS	3.8871***	-0.2872*	4.0129***
	(8.6592)	(-1.9096)	(8.9414)
GRO	0.3635*	-0.0095	0.3345
	(1.7501)	(-0.2861)	(1.6276)
OVE	-0.0185*	-0.0023	-0.0172*
	(-1.9474)	(-0.6389)	(-1.8901)
LEV	1.8426***	0.6189***	1.6141***
	(6.6520)	(3.4537)	(5.6595)
AGE	0.5358***	-0.0608***	0.5671***
	(8.2178)	(-2.7265)	(8.8471)
Constant	10.3625***	-2.3154***	11.0138***
	(9.1720)	(-5.8677)	(10.2103)
Year	Control	Control	Control
Ind	Control	Control	Control
R-squared	0.3326	0.1419	0.3386
N	1882	1882	1882

### Heterogeneity analysis

#### Company category

China’s listed energy companies consist of state-owned companies and non-state-owned companies. They differ in management models, governance approaches, and social statuses. Their intrinsic motivations and implementation modes for ESG responsibilities and obligations vary^61^. State-owned companies focus on value enhancement and public welfare attributes in their development. They assume public and societal responsibilities and obligations, facing high levels of supervision and public concern from society and media. This drives stronger enthusiasm for ESG governance among them. Non-state-owned companies prioritize profit and value in their development. They engage in ESG governance to meet stakeholder requirements and enhance company value. However, their resources are constrained. Excessive ESG investment beyond reasonable limits may constrain their development. Additionally, resource advantages and social statuses differ between state-owned and non-state-owned companies^62^. State-owned companies, as national asset institutions, maintain closer ties with the government. They receive greater policy support and financial subsidies from the government compared to non-state-owned companies. State-owned companies are larger in scale and higher in social status than non-state-owned ones. These factors drive state-owned companies to invest more in ESG responsibilities and obligations. In summary, ESG performance differs between state-owned and non-state-owned companies. Whether ESG performance’s positive impact on “going global” performance varies across company types requires further investigation.

Table 9 presents the heterogeneity analysis results by company category. The findings reveal that ESG exerts a significant positive effect on listed energy companies’ “going global” performance across both categories. However, the magnitude of the impact varies by company category. Column (1) shows the ESG coefficient for non-state-owned companies is 0.6032, which is significant at the 1% level, indicating non-state-owned companies significantly enhance their “going global” performance through improved ESG practices. Column (2) reveals a more pronounced effect, with an ESG coefficient of 0.9666 and significant at the 1% level, suggesting state-owned companies have a stronger positive impact on “going global” performance when enhancing ESG performance compared to non-state-owned peers. This discrepancy is attributed to state-owned companies’ access to policy support and stronger international image.

**Table 9:** Heterogeneity analysis results of company category.

Variables	(1)	(2)
	Overseas	Overseas
ESG	0.6032***	0.9666***
	(3.4787)	(6.4056)
TAT	0.7375***	0.9284***
	(4.2288)	(3.3252)
TOP10	-0.2071	0.9235**
	(-1.1385)	(2.4012)
SIZE	4.1325***	3.2521***
	(4.0666)	(4.0555)
FAS	3.8181***	3.4076**
	(8.0670)	(2.3779)
GRO	0.4749*	0.0427
	(1.8522)	(0.1615)
OVE	-0.0195	-0.0785**
	(-1.4338)	(-2.4080)
LEV	2.1897***	1.5805*
	(7.4938)	(1.8561)
AGE	0.4283***	0.9709***
	(7.0677)	(6.0118)
Constant	12.1456***	9.9708***
	(7.5453)	(6.1238)
Year	Control	Control
Ind	Control	Control
R-squared	0.2831	0.5221
N	1342	540

#### Energy type

China’s new energy consumption has maintained a long-term growth trend in recent years, aligning with green economy and sustainable development. It boasts advantages like low pollution, large reserves, high efficiency, and low consumption, giving new energy companies natural ESG performance advantages^63^. Traditional energy companies, due to inherent high pollution and carbon emissions, perform weaker environmentally than new energy companies, prompting stronger green development behaviors. Traditional energy companies actively promote innovation-driven approaches and energy transformation, narrowing the ESG performance gap with new energy companies over time. Currently, ESG performance differs between the two sectors. This paper references key energy sector research and aligns with the main business of sample companies, further dividing samples into traditional and new energy companies. Whether ESG performance’s positive impact on “going global” performance varies across energy types requires investigation. Results are shown in Table 10.

Column (1) shows the ESG coefficient for traditional energy companies is 0.5775, which is significant at the 1% level. Column (2) reports that the ESG coefficient for new energy companies is 0.7335, which is significant at the 1% level. This indicates ESG effectively enhances “going global” performance across both traditional and new energy companies. The higher coefficient for new energy companies suggests their “going global” performance is more responsive to ESG. This reflects stronger reliance on sustainable development principles and alignment with international market preferences among new energy companies. The mediation mechanism is shaped by both company category and energy type. The impact of ESG is more significant for different company categories.

**Table 10:** Heterogeneity analysis results of energy type.

Variables	(1)	(2)
	Overseas	Overseas
ESG	0.5775***	0.7335***
	(3.8144)	(4.0280)
TAT	0.8985***	2.5443***
	(4.2664)	(6.6051)
TOP10	0.2065	0.4214
	(1.0766)	(1.5147)
SIZE	2.7698***	4.2364***
	(4.7170)	(4.4088)
FAS	3.9471***	0.7227
	(4.2049)	(1.3116)
GRO	0.0159	-0.3915
	(0.0611)	(-1.5908)
OVE	-0.0536***	-0.0005
	(-4.2205)	(-0.0413)
LEV	0.5276	2.5386***
	(1.0456)	(4.2093)
AGE	0.7102***	0.6210***
	(8.9446)	(6.0585)
Constant	13.1372***	10.2766***
	(10.3516)	(7.1960)
Year	Control	Control
Ind	Control	Control
R-squared	0.3923	0.4588
N	1059	823

#### Host country

Due to variations in local environmental and social compliance requirements across host countries, the competitive advantages derived from the ESG performance of China’s listed energy companies may exhibit heterogeneity across different markets. A company ranked at the top for ESG domestically might only achieve a marginal passing grade in countries with stringent environmental standards. Conversely, in countries with lower environmental thresholds, a company with a modest domestic ESG score might possess a “dimensionality reduction” competitive advantage^64^. In 2013, China proposed the Belt and Road Initiative (BRI). The countries along the BRI are predominantly developing countries characterized by abundant traditional energy reserves, relatively weak infrastructure, and environmental policy stringency that is generally far lower than that of developed countries^65^. In developing countries, governments may prioritize economic growth and extraction efficiency, maintaining relatively lax regulations on environmental protection. The massive investments in green innovation by Chinese companies may fail to translate into a competitive advantage; instead, they might even lead to a disadvantage due to increased operational costs^66^. Consequently, based on company annual reports and the BRI company directory, we categorized the total sample into two sub-samples based on the geographic focus of their overseas operations: “High-BRI Companies” and “Non-BRI Companies”. This classification allows us to examine whether heterogeneity exists in the positive impact of ESG performance on “going global” performance, as well as in the mediating role of green innovation across different host markets.

Comparing Columns (1) and (4) of the regression results in Table 11, it is evident that ESG performance exerts a significant positive influence on “going global” performance across both samples, indicating that ESG advantages can effectively translate into performance gains. However, the magnitude of this impact varies depending on the host markets. The ESG regression coefficient for the Non-BRI sample is 0.8214, which is markedly higher than the 0.4532 observed for the High-BRI sample. This suggests that in markets with stringent environmental policies, the marginal performance premium for improving ESG performance is substantially higher. In contrast, while ESG performance still facilitates “going global” performance in markets with laxer regulations, its promotional effect is only approximately 55% of that found in stringent markets.

The disparity in the mediating role of green innovation across host markets is quantified through the three-stage regression results in Table 11. The empirical results show that green innovation passes the mediation effect test in both samples. In the Non-BRI sample, Column (2) shows that ESG significantly drives green innovation with a coefficient of 0.4782, and Column (3) shows that green innovation positively impacts “going global” performance with a coefficient of 0.2934. Green innovation accounts for approximately 17.1% of the total effect’s performance increment in this sample. In the High-BRI sample, Columns (5) and (6) indicate that the mediating path for green innovation remains significant with a coefficients of 0.2431 and 0.1328 respectively, but its explanatory power regarding the total effect drops to approximately 7.1%. The green innovation regression coefficient for the Non-BRI sample is approximately 2.2 times that of the High-BRI sample. These data suggest that while ESG and green innovation serve as universal competitive advantages for China’s listed energy companies “going global”, their efficiency in promoting performance is moderated by the environmental institutional frameworks and energy structures of the host countries.

**Table 11:** Heterogeneity analysis results of host country.

Variables	(1)	(2)	(3)	(4)	(5)	(6)
	Overseas	GRE	Overseas	Overseas	GRE	Overseas
ESG	0.8214∗∗∗	0.4782∗∗∗	0.6125∗∗∗	0.4532∗∗∗	0.2245∗∗	0.4358∗∗∗
	(5.6841)	(5.9241)	(5.2104)	(3.1245)	(2.5681)	(3.7214)
GRE			0.2934∗∗∗			0.1512∗∗
			(4.3215)			(2.1852)
TAT	0.8124∗∗∗	-0.0125	0.8432∗∗∗	0.8432∗∗∗	-0.0104	0.8214∗∗∗
	(3.1023)	(-0.1852)	(3.9241)	(3.9241)	(-0.1425)	(3.8647)
TOP10	-0.1654	-0.2451∗∗∗	0.2872	-0.1082	-0.1842∗∗	0.1654
	(-1.3214)	(-7.4123)	(1.4236)	(-1.0123)	(-2.1023)	(0.8641)
SIZE	3.5412∗∗∗	1.4258∗∗∗	3.5412∗∗∗	4.1236∗∗∗	1.2542∗∗∗	4.1236∗∗∗
	(3.8645)	(5.3214)	(3.8645)	(5.4231)	(4.8641)	(5.4231)
FAS	3.2145∗∗∗	-0.2847	3.2145∗∗∗	4.3124∗∗∗	-0.2105	4.3124∗∗∗
	(5.9241)	(-1.8641)	(5.9241)	(4.5641)	(-1.1023)	(4.5641)
GRO	0.2874	-0.0102	0.2874	0.5214∗∗	0.0124	0.5214∗∗
	(1.4236)	(-0.2987)	(1.4236)	(2.1245)	(0.2458)	(2.1245)
OVE	-0.0105	-0.0031	-0.0105	-0.0425∗∗∗	-0.0112	-0.0425∗∗∗
	(-0.7214)	(-0.6124)	(-0.7214)	(-2.9241)	(-0.3541)	(-2.9241)
LEV	1.3258∗∗∗	0.6214∗∗∗	1.3258∗∗∗	1.7142∗∗∗	0.5874∗∗∗	1.7142∗∗∗
	(3.8647)	(3.5412)	(3.8647)	(3.2145)	(3.2145)	(3.2145)
AGE	0.4872∗∗∗	-0.0614∗∗∗	0.4872∗∗∗	0.5412∗∗∗	-0.0402	0.5412∗∗∗
	(6.2145)	(-2.8641)	(6.2145)	(4.3124)	(-1.2145)	(4.3124)
Constant	11.2145∗∗∗	-2.4102∗∗∗	11.2145∗∗∗	9.5412∗∗∗	-1.8852	9.5412∗∗∗
	(8.5412)	(-6.0125)	(8.5412)	(6.3214)	(-1.5102)	(6.3214)
Year	Control	Control	Control	Control	Control	Control
Ind	Control	Control	Control	Control	Control	Control
R-squared	0.3542	0.1752	0.3842	0.2612	0.1065	0.3012
N	565	565	565	1317	1317	1317

## Discussion and implications

Research related to ESG has an explosive growth in recent years. Extant literature focuses primarily on ESG information disclosure, ESG rating divergence, and the impact of ESG performance on economic consequences including company value and business performance^4–6^. At present, the discussion of companies’ ESG on the “going global” performance is a lack of research, especially for Chinese energy companies. This paper discusses the connection between ESG performance and “going global” performance. Based on financial data (2013–2023) of Chinese A-share listed energy companies, the research indicates that, good ESG performance of companies can significantly enhance their “going global” performance. Our findings are broadly consistent with international evidence documenting a positive ESG and international performance link^67–69^, while differences may arise from disclosure regimes, different industry, and rating methodologies. Meanwhile, enhancing company reputation and promoting green innovation have a mediating effect. They are potential channels through which ESG performance affects the “going global” performance for companies. In addition, company category and energy type show significant heterogeneity in this impact for listed energy companies.

The “going global” performance is also overseas business performance. In many current studies, overseas foreign direct investment^19,20,70^ or export amounts^24,71,72^ are used to measure this performance. On this basis, this paper uses the overseas revenue indicator of companies to measure it, to obtain meaningful research conclusions. Many studies choose all listed companies as research samples, which may mask differences between industries. But this paper focuses on energy companies. They possess the distinct characteristics of high pollution, high emissions, and high energy consumption. The research finds that, through promoting green innovation, ESG performance of listed energy companies can be improved, to enhance their “going global” performance. This study has practical significance, and is in line with the historical background. It can provide support ideas into ESG for Chinese listed energy companies to “go global”.

### Theoretical implications

This study enriches the research on the factors influencing the “going global” performance. In the past, scholars focused on factors such as company productivity and innovation management, when most scholars discussed overseas business performance. Less attention was paid to the performance of companies in ESG. The international competitiveness of companies is affected by ESG performance, as an important factor, with increasing global attention to sustainable development. This paper is based on the signal theory and sustainable development theory, to explore how ESG performance affects the “going global” performance, and to verify its impact mechanism. The study provides evidence of Chinese companies in specific industries, and the results enrich existing research findings in ESG research field.

This study enriches the research on the economic consequences due to ESG performance. There are few research results on this topic, and the conclusions are inconsistent. One viewpoint is that the costs invested by practicing ESG will inhibit business performance in the short-term for companies “going global”. However, in the long-term, good ESG performance will have a positive effect on “going global” performance. The other viewpoint is that, if companies can demonstrate advantages in ESG performance, it can effectively lower the entry threshold of the host country when “going global”. This allows for faster expansion into overseas markets, improving the companies’ “going global” efficiency. This study quantitatively evaluates the impact of ESG performance on the economic consequences for companies “going global”, and proposes the conclusions. These findings enrich the theoretical research on ESG performance and “going global” performance.

This paper innovatively explores the mechanism by which ESG performance affects “going global” performance. There are few research results on this impact. The research mainly focuses on company overseas investment, mergers, and acquisitions. This study is conducted from the perspectives of the signal theory, reputation theory, and sustainable development theory. We find that, two mechanism paths also exist: corporate reputation and green innovation, in addition to the financing constraint paths validated by existing research. ESG policy pressure runs through the entire value chain of the industry, especially for the energy industry. This study clarifies the impact mechanism of ESG performance on “going global” performance, and enriched existing research results.

### Practical implications

The global market is paying more attention to environmental protection and social responsibility. Strict environmental protection laws, labor rights laws and governance standards are being introduced by more and more countries and regions. Chinese companies face more severe challenges in “going global”. In term of practical implications, this study can provide ESG breakthrough ideas in the “going global” development and green transformation for Chinese listed energy companies. At present, the listed energy companies face pain points in their “going global” process, including green barriers, market transformation, political risks, compliance with environmental regulations, and company reputation. ESG is no longer just a company social responsibility, but also core competitiveness for its sustainable development in the global market, from a practical perspective. A company’s excellent ESG performance can effectively alleviate the pain it faces when “going global”. For example, by improving environmental standards, strengthening social responsibility and improving governance structures, companies can enhance their competitiveness and market access capabilities in the international market. In addition, these measures can reduce external risks and attract more investment. Finally, these improvements will promote the growth of overseas business and help “going global”.

Through comprehensive analysis of theories and current situations, this paper empirically confirms the positive impact of ESG advantages in promoting the “going global” performance for listed energy companies. The empirical evidence helps boost listed energy companies’ attention to their ESG performance. The study advocates that while pursuing current benefits, the listed energy companies also pay attention to performance in terms of environment, social responsibility and company governance. The listed energy companies will adhere to the concept of sustainable development, and ultimately achieve improved “going global” performance. Meanwhile, it is also beneficial for the country to practice the concept of green and high-quality development, conveying the image of a responsible company and a major power to the international community.

In summary, our findings suggest that ESG is not merely a compliance requirement but a strategic capability that can enhance listed energy companies’ international market access and overseas business growth by improving reputation and fostering green innovation. Accordingly, managers and policymakers should jointly promote credible ESG governance and disclosure to reduce cross-border information frictions and mitigate regulatory and reputational risks during internationalization.

## Limitations and future research

However, this research has also limitations. The study sample is not completely comprehensive. While studying, we found that some companies’ “going global” performance data were discontinuous, affected by COVID-19. These data were excluded, resulting in a decrease in the study sample. Due to the limitation of the proportion of China’s listed energy companies going global, the sample size is also relatively small. Nowadays, ESG information disclosure of Chinese companies is showing an accelerated development trend, also including policy system and market practice. Chinese companies will disclose more comprehensive ESG data. The study sample will be more abundant on this topic. In the future, we will conduct more refined research. Richer measures of internationalization would allow a closer match between company ESG performance and host-country market conditions. These measures include more comprehensive company ESG disclosure information, as well as more company performance indicators that can be considered for inclusion in the “going global” performance. The non-linear relationship between ESG performance and “going global” performance will be further considered and checked.

## Conclusions

This paper uses literature review, theoretical analysis, and empirical analysis as research methods. The sustainable development theory, signal transmission theory, and reputation theory are the foundations of the research. The listed energy companies from 2013 to 2023 are the research objects. This paper focuses on the impact of ESG performance of listed energy companies on their “going global” performance, as the research topic. The impact and mechanism were tested by empirical analysis. This paper obtains the following conclusions:

ESG performance helps promote the improvement of “going global” performance for the listed energy companies. The baseline regression results show that it has a significant positive impact, with a coefficient of 0.7816 and the significant at 1% level. This paper adopts the methods of changing sample period and replacing explanatory variable, to test the results robustness, and to address the issue of selection bias in the samples. The conclusions remain valid, enhancing the reliability of research results.

The mechanism analysis indicates that company reputation and green innovation have a mediation effect, verifying the hypotheses. Good ESG performance enhances company reputation, thereby improving the “going global” performance of listed energy companies. Good ESG performance directly improves the “going global” performance of listed energy companies, also indirectly expands their competitiveness and attractiveness in the international market, by promoting green innovation.

The heterogeneity analysis indicates that superior ESG performance significantly enhances “going global” performance of listed energy companies across different company categories, energy types and host markets. Specifically, the promoting effect is more pronounced for state-owned companies than non-state-owned ones, and for new energy companies compared to traditional ones. Furthermore, while ESG benefits companies in both host markets, those in non-Belt and Road Initiative markets exhibit higher sensitivity to ESG and a stronger mediating role of green innovation.

## Data Availability

Data is available on request to the authors.
